# Interobserver agreement on landmark and flexure identification in colon capsule endoscopy

**DOI:** 10.1007/s10151-023-02789-z

**Published:** 2023-04-10

**Authors:** B. Schelde-Olesen, T. Bjørsum-Meyer, A. Koulaouzidis, M. M. Buijs, J. Herp, L. Kaalby, G. Baatrup, U. Deding

**Affiliations:** 1 Department of Clinical Research, University of Southern Denmark, Odense, Denmark; 2 Department of Surgery, Odense University Hospital, Baagoes Alle 31, 5700 Svendborg, Denmark; 3 Department of Social Medicine and Public Health, Pomeranian Medical University, 70-204 Szczecin, Poland; 4 Applied AI and Data Science Group, Mærsk-Mc-Kinney Møller Institute, Faculty of Engineering, University of Southern Denmark, Odense, Denmark; 5 CAI-X (Centre for Clinical Artificial Intelligence) University of Southern Denmark and Odense University Hospital, Odense, Denmark; 6 Department of Medicine, Odense University Hospital, Svendborg, Denmark

**Keywords:** Colon capsule endoscopy, Landmark, Localization, Interobserver agreement

## Abstract

**Purpose:**

When an optical colonoscopy is carried out, Scope Guide can assist the endoscopist in determining the localization. In colon capsule endoscopy (CCE), this support is not available. To our knowledge, the interobserver agreement on landmark identification has never been studied. This study aims to investigate the interobserver agreement on landmark identification in CCE.

**Methods:**

An interobserver study was carried out comparing the landmark identification (the ileocecal valve, hepatic flexure, splenic flexure, and anus) in CCE investigations between an external private contractor and three in-house CCE readers with different levels of experience. All CCE investigations analyzed in this study were carried out as a part of the Danish screening program for colorectal cancer. Patients were between 50 and 74 years old with a positive fecal immunochemical test (FIT). A random sample of 20 CCE investigations was taken from the total sample of more than 800 videos.

**Results:**

Overall interobserver agreement on all landmarks was 51%. Interobserver agreement on the first cecal image (ileocecal valve), hepatic flexure, splenic flexure, and last rectal image (anus) was 72%, 29%, 22%, and 83%, respectively. The overall interobserver agreement, including only examinations with adequate bowel preparation (*n* = 16), was 54%, and for individual landmarks, 73%, 32%, 24%, and 85%.

**Conclusion:**

Overall interobserver agreement on all four landmarks from CCE was poor. Measures are needed to improve landmark identification in CCE investigations. Artificial intelligence could be a possible solution to this problem.

## Introduction

Optical colonoscopy (OC) is considered the cornerstone of colorectal cancer (CRC) diagnostics. In 2006, the first-generation PillCam™ colon capsules (Medtronic, Minneapolis, Minnesota, USA) were introduced as an alternative diagnostic modality. It was soon replaced by the second generation, which is currently used worldwide. Colon capsule endoscopy (CCE) has a diagnostic accuracy for colorectal polyps similar to OC [1], with a low complication rate and high patient-reported tolerability [2]. However, CCE lacks biopsy and/or polypectomy capabilities. Therefore, accurate lesion localization in CCE is critical in planning subsequent therapeutic interventions. A correctly reported localization of lesions in CCE will aid the endoscopist in the subsequent OC, whereas an incorrect localization can be an obstacle to an effective procedure.

**Fig. 1:**
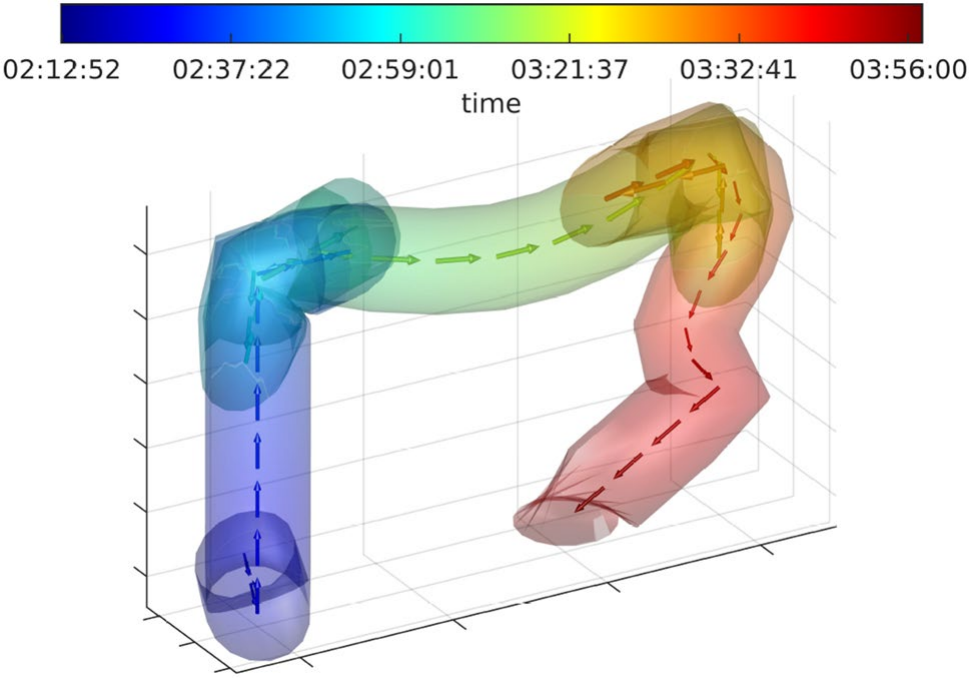
Illustration of capsule movement Illustration of the estimated path taken by a capsule. On the basis of the algorithm by Herp et al. [3], 10,000 capsule paths are estimated with varying parametrization of the colon radius and capsule sample frequency (based on one CCE video). The mean path is indicated as arrows color-coded according to the elapsed time, indicating the flow of information between consecutive frames, i.e., the direction in which the capsule moves. The tube surrounding the path contains 95% of all 10,000 estimated paths

Currently, CCE videos are previewed by readers to determine the landmarks that serve as reference points for the localization of lesions. However, the uncontrollable capsule movement and the possibility of the capsule passing the landmarks multiple times make it difficult for the reader to keep track of the capsule’s orientation. Figure 1 illustrates the estimated path taken by a single specific capsule based on the AI algorithm developed by Herp et al. [3]. Despite the challenges, many regard the reported localization as reasonably accurate. The information on localization is utilized in research and clinical settings, even though the interobserver agreement on landmark identification, to our knowledge, has never been studied. This study aimed to investigate the interobserver agreement on landmark identification in CCE.

## Materials and methods

### Study design

This is an interobserver study comparing the landmark identification in CCE investigations between a group of CCE readers employed at an external private contractor and three in-house CCE readers with different levels of experience.

### CCE videos

All CCE videos were prospectively collected as part of an ongoing study, CAREforCOLON 2015 (CfC2015), that investigates the possibility of implementing CCE in the Danish CRC screening program [4, 5]. All participants were 50 to 74 years old with a positive fecal immunochemical test (FIT > 100 ngHb/mL buffer) [6]. For this study, a random sample of 20 CCE investigations was drawn from a total of 856 videos available using SAS software version 9.4 (SAS Institute Inc., SAS 9.4., Cary, NC, USA). Experienced CCE readers from an external private contractor [Corporate Health International (CHI), Hamburg, Germany] evaluate the CCE videos and generate a report. These reports include timestamps pinpointing the four colonic landmarks.

### CCE readers

The group of in-house CCE readers comprised three clinicians with different experience levels in CCE. One is considered an expert (AK), having evaluated more than 2000 CCE investigations before this study; another is considered experienced (MMB) with limited CCE reading experience (84 CCEs); and one is a novice without previous experience in CCE reading. All three are experienced endoscopists. The group of readers from the external private contractor represented differing experience levels but were managed by CCE experts responsible for the final report. These CCE experts are experienced medical doctors with expertise in capsule endoscopy. Details regarding the specific level of experience within the CCE reader group at the external private contractor are not available to us. All CCE readers involved in reporting for this study went through a structured course in the beginning of their employment followed by a period of supervised CCE reporting.

### Landmarks

**Fig. 2:**
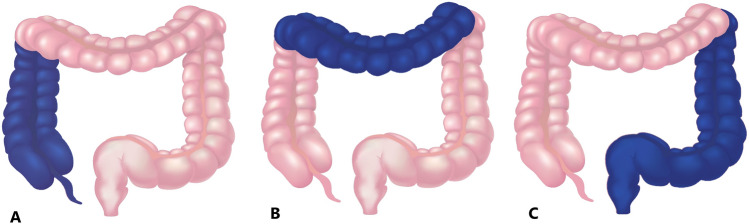
Segments of the large bowel. **a** Right colon. **b** Transverse colon. **c** Left colon. Image from Colourbox

**Fig. 3:**
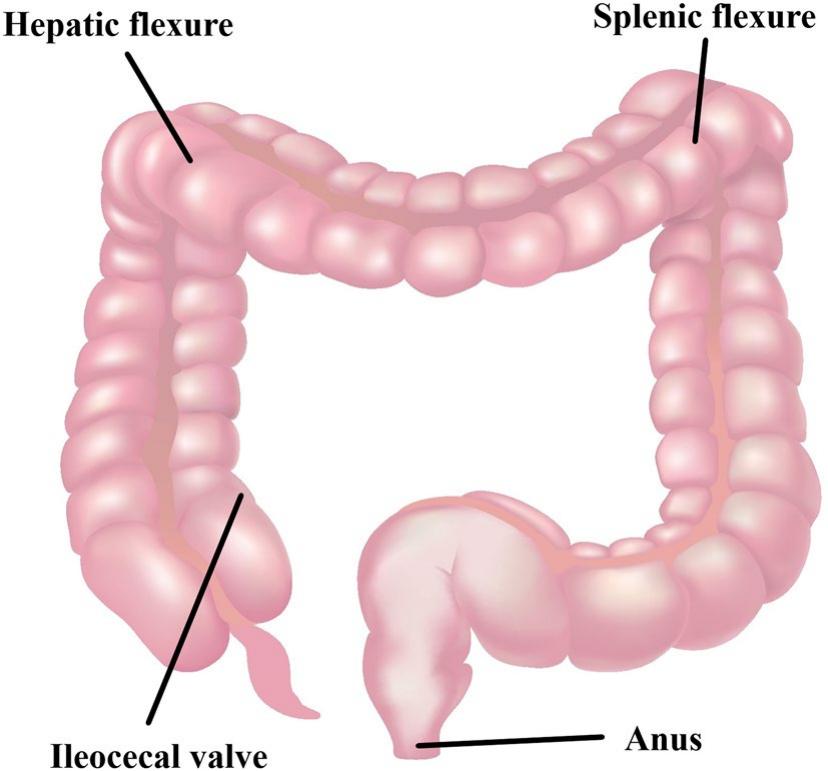
Colonic landmarks. Image from Colourbox

For each CCE investigation, we divided the large bowel into three segments: the right, the transverse, and the left colon (Fig. 2). The ileocecal valve, hepatic flexure, splenic flexure, and anus created the landmarks used to determine the segments (Fig. 3). The ileocecal valve was defined as the first image when the capsule entered the colon (first cecal image) and the anus as the last image before the capsule was excreted from the rectum (last rectal image).

The first cecal and last rectal images were reported as specific timestamps. The agreement between readers on the first cecal and last rectal images was determined as identical timestamps ± 1 s. The margin of 1 s was given to leave room for small deviations in the interpretation of what constitutes the first cecal image and the last rectal image and thereby reduce the risk of false negative matches. However, the margin was kept short to avoid false positive matches, as the capsule can move rapidly through a segment in only seconds.

The hepatic and splenic flexure were reported as intervals and could be reported more than once in case of backward progression of the capsule. For agreement on flexure identification, intervals reported by the readers should have a complete or partial overlap. If a reader noted several passages, only an overlap in one of the passages was necessary for the agreement to be present. The external private contractor reported only one timestamp for each flexure instead of an interval. Therefore, agreement with the in-house CCE readers was defined as a timestamp included in an interval given by the CCE readers.

### Data collection

The in-house CCE readers used the online platform PillCam™ Web Software (Given Imaging Inc, USA) to display the CCE videos. Both oral and written instructions on how to record the landmarks were given to the three readers, and they were supplied with a digital form to ensure uniform reporting. The written instructions are available in Appendix A. The in-house readers were blinded to the report from the external private contractor and each other.

### Statistical analysis

Interobserver agreement was determined as the percent agreement (i.e., proportions) on the 20 videos between the four readers. The agreement was assessed manually on the basis of previously described definitions of agreement. The timestamps identified by the in-house readers were compared pairwise to the external private contractor and each other. We calculated pairwise agreements between readers overall and per landmark. Additionally, overall agreement and per landmark agreement between all readers were calculated. A sensitivity analysis was performed after excluding videos with unacceptable bowel preparation to reduce the effect of bowel cleansing quality on identifying the landmarks. Statistical analyses were performed using SAS software version 9.4 (SAS Institute Inc. SAS 9.4. Cary, North Carolina, USA).

## Results

When we initiated this study, 856 consecutive CCE investigations were available in the CfC2015 database. Fourteen investigations (1.6%) were excluded as a result of capsule retention in the stomach, small bowel, or technical errors in the video recording process. A random sample of 20 videos was drawn from the remaining 842 CE investigations. One single timestamp was missing from the external private contractor (splenic flexure, CCE video no. 20, Appendix B).

The interobserver agreement on the four individual landmarks and all landmarks combined is presented in Table 1. Results from the sensitivity analysis of CCE videos with sufficient bowel preparation (*n* = 16) are presented in Table 2. An overview of all results is available in Appendix B, including the agreement on each specific CCE investigation.

**Table 1:** Interobserver agreement between the individual CCE readers and overall agreement on all and individual landmarks including all CCE investigations (*n* = 20)

	Expert	Experienced	Novice	Overall agreement
**All landmarks**				
External contractor	50%	55%	39%	51%
Expert	–	64%	48%	
Experienced	–	–	54%	
**First cecal image**				
External contractor	65%	65%	55%	72%
Expert	–	90%	75%	
Experienced	–	–	80%	
**Hepatic flexure**				
External contractor	30%	25%	5%	29%
Expert	–	65%	35%	
Experienced	–	–	15%	
**Splenic flexure**				
External contractor	5%	40%	20%	22%
Expert	–	10%	5%	
Experienced	–	–	50%	
**Last rectal image**				
External contractor	100%	90%	75%	83%
Expert	–	90%	75%	
Experienced	–	–	70%

**Table 2:** Interobserver agreement between the individual CCE readers and overall agreement on all and individual landmarks including only CCE investigations with sufficient bowel preparation (*n* = 16)

	Expert	Experienced	Novice	Overall agreement
**All landmarks**				
External contractor	52%	56%	41%	54%
Expert	–	66%	50%	
Experienced	–	–	59%	
**First cecal image**				
External contractor	69%	69%	56%	73%
Expert	–	88%	75%	
Experienced	–	–	81%	
**Hepatic flexure**				
External contractor	38%	25%	6%	32%
Expert	–	69%	38%	
Experienced	–	–	19%	
**Splenic flexure**				
External contractor	0%	38%	25%	24%
Expert	–	13%	6%	
Experienced	–	–	63%	
**Last rectal image**				
External contractor	100%	94%	75%	85%
Expert	–	94%	75%	
Experienced	–	–	75%	

The overall interobserver agreement for all landmarks was 51%, and the overall agreement on the first cecal image, hepatic flexure, splenic flexure, and last rectal image was 72%, 29%, 22%, and 83%, respectively. The best interobserver agreement was detected between the in-house expert and experienced reader (64%), and the lowest agreement was between the external private contractor and the novice reader (39%). However, a general trend of higher agreement between more experienced readers was not seen. Interobserver agreement was as low as 5% for hepatic and splenic flexures and as high as 100% for the last rectal image (Table 1). The overall interobserver agreement, including only examinations with sufficient bowel preparation (*n* = 16), was 54%. The overall agreement on the first cecal image, hepatic flexure, splenic flexure, and last rectal image in those 16 examinations was 73%, 32%, 24%, and 85%, respectively (Table 2).

## Discussion

The overall interobserver agreement on landmark identification in this study was 51%, which improved slightly when excluding CCE videos with unacceptable bowel preparation. No guidelines exist on what an acceptable agreement is. We researched the literature but could not find other articles discussing agreement on landmark identification in CCE. Still, we consider 51–54% a poor agreement. The agreement on identifying the first cecal and last rectal images was distinctly better than identifying the two flexures. This can be explained by the fact that the confining colonic landmarks (the ileocecal valve and the anal valve) are characterized by an apparent change in the imaged mucosal structures or the mucosa and the excretion environment. However, one would expect the agreement to be close to 100% since it should be no more than identifying the first and last image of the colon. This could be caused by different interpretations of the instructions by the in-house readers, highlighting the need for a consensus on CCE reporting. It would be interesting to assess the individual CCE videos to find out why the overall agreement only reaches 73% on the first cecal image and 85% on the last rectal image.

Still, when dividing the colon into different segments using the flexures, there is no discernible difference in the mucosal appearance from one segment to another. Theoretically, the identification of the transverse colon should be simple, based on the triangular lumen. However, our results did not confirm this. This could be due to the lack of insufflation of the colon in CCE compared to colonoscopy, where the shape of the colon is more pronounced. Additionally, in colonoscopy, the endoscopist controls the orientation of the scope, which enables easier identification of the luminal shape. Although OC possesses some advantages in landmark localization due to the controlled movement and possibility of ScopeGuide assistance, previous studies have shown that lesion localization in OC is not optimal. A meta-analysis reporting on preoperative CRC localization showed an incidence of localization errors in OC of 15.4% [7]. Evidently, OC, which we consider the gold standard for the detection of colorectal neoplasia, is not flawless in localization either.

Since the clinical introduction of capsule endoscopy in 2001, several systems to support manual analysis have been suggested. However, most capsule systems are developed for small-bowel investigations. The different techniques for localization of the capsule and possible lesions include software using radiofrequency transmission [8], capsule-odometry [9], and artificial intelligence (AI) algorithms [10]. All were developed to report a precise localization of the capsule in the gastrointestinal tract. Because CCE in routine clinical practice is relatively new, no localization system is currently implemented in the assessment of the videos. Herp et al. proposed an AI algorithm that identifies the shape of the colon and estimates the camera capsule’s movement based on CCE video material [3]. The study showed that the accuracy of the capsule localization reported by the AI algorithm decreases with increasing distance to a known starting point (in this study, the anus).

If we can identify the flexures consistently, we can reset the accumulated inaccuracy in capsule location when the capsule passes the landmarks. To train an algorithm properly, we need a high validity of the ground truth information feeding it. This cannot be accomplished as long as CCE readers cannot identify the landmarks consistently and in agreement. Expert reader consensus may be the best ground truth moving forward. As CCE reading is very time-consuming and costly, the future of CCE reading must entail the support of AI [11, 12]. In both manual and AI-supported reading, we can only accurately locate lesions or evaluate bowel cleansing by segment once the agreement on landmark identification has improved. Still, the current uncertain localization is used in clinical settings, causing difficulties for the endoscopist to locate lesions at the following therapeutic colonoscopy. This is a definite problem, as unnecessary time spent searching for CCE-reported findings in the wrong bowel segment could cause frustration to the endoscopist and undue discomfort for the patient. Furthermore, the lack of precision in localizing CCE findings is an obstacle to research in this area, and we, therefore, risk drawing faulty conclusions based on incorrect data.

We acknowledge some limitations to this study. One is the discrepancy in how the different landmarks were reported between the in-house readers and the external private contractor. However, we do see the need for comparing the evaluation by the in-house readers to the assessment used in clinical practice, here represented by the external private contractor. Missing data was minimal, as only one single timestamp was missing from the entire data collection. The match definition for the first cecal image and the last rectal image of ± 1 s will undoubtedly affect the percent agreement. A more considerable margin for a match could increase the agreement. We decided on this narrow margin to avoid false positive matches, as we know that the capsule can travel through an entire segment in only a few seconds. The novice reader did not receive any formal training in CCE reading. However, this did not seem to affect the agreement with the other readers except for identifying the last rectal image.

## Conclusion

Interobserver agreement on landmark identification between CCE readers was low in this study, although better for the first cecal image and the last rectal image as compared to flexure identification. Ways of increasing the agreement must be developed to improve the accuracy of lesion localization. This is necessary to develop and train AI for landmark identification properly.

## Data Availability

As a result of the nature of this research, patients did not agree for their data to be shared publicly. Therefore supporting data is not available.

## Appendix A

### Written instructions to capsule readers

- Timestamps are marked as hour:minute:second (e.g., 01:01:01).
- First cecal image: timestamp marking when the capsule enters the colon.
- Last rectal image: timestamp marking the last image before the capsule is excreted from the rectum.
- Hepatic flexure:
  - First image: timestamp marking when the capsule enters the hepatic flexure.
  - Last image: timestamp marking when the capsule leaves the hepatic flexure.
- Splenic flexure:
  - First image: timestamp marking when the capsule enters the splenic flexure.
  - Last image: timestamp marking when the capsule leaves the splenic flexure.
- If the capsule drifts between segments and the flexure is seen more than once the columns marked respectively *hepatic* or *splenic flexure 2. 3. and 4. passage* are filled out.

## Appendix B

Interobserver agreement on landmarks in individual CCE investigations
